# Statistical validation of megavariate effects in ASCA

**DOI:** 10.1186/1471-2105-8-322

**Published:** 2007-08-30

**Authors:** Daniel J Vis, Johan A Westerhuis, Age K Smilde, Jan van der Greef

**Affiliations:** 1 BioSystems Data Analysis group, Swammerdam Institute for Life Science, University of Amsterdam, The Netherlands; 2 TNO Quality of Life, Zeist, The Netherlands

## Abstract

**Background:**

Innovative extensions of (M) ANOVA gain common ground for the analysis of designed metabolomics experiments. ASCA is such a multivariate analysis method; it has successfully estimated effects in megavariate metabolomics data from biological experiments. However, rigorous statistical validation of megavariate effects is still problematic because megavariate extensions of the classical F-test do not exist.

**Methods:**

A permutation approach is used to validate megavariate effects observed with ASCA. By permuting the class labels of the underlying experimental design, a distribution of no-effect is calculated. If the observed effect is clearly different from this distribution the effect is deemed significant

**Results:**

The permutation approach is studied using simulated data which gave successful results. It was then used on real-life metabolomics data set dealing with bromobenzene-dosed rats. In this metabolomics experiment the dosage and time-interaction effect were validated, both effects are significant. Histological screening of the treated rats' liver agrees with this finding.

**Conclusion:**

The suggested procedure gives approximate p-values for testing effects underlying metabolomics data sets. Therefore, performing model validation is possible using the proposed procedure.

## 1 Background

In life science research many measuring tools emerged in recent years. These tools give a coarse profile of biological classes such a transcripts (transcriptomics), proteins (proteomics) and metabolites (metabolomics). This paper focuses on the field of metabolomics; the comprehensive quantitative and qualitative analysis of all small molecules of cells, body fluids, and tissues. The mix of hypothesis and discovery driven omics-experiments create novel biostatistical challenges noted since combining pattern recognition and body fluid profiling in the early eighties [1]. Interpreting the multivariate metabolomics results means integrating biological knowledge with possible contributing metabolites.

Metabolomics data sets comprise hundreds of metabolites measured in typically tenths of samples. Multivariate statistics on data that have fewer samples than metabolites is cumbersome. Usually there is an experimental design underlying the metabolomics data sets. The obvious technique for analyzing such data, Multivariate Analysis of Variance (MANOVA) [2] cannot deal with data that consists of more metabolites than samples.

The recent introduction of ANOVA-based extensions of multivariate data analysis methods may open new angles to analyze metabolomics data. These methods aim to analyze designed experiments with more measured metabolites than samples. Among the new methods are ANOVA-principal component analysis (PCA), principal response curves (PRC) and ASCA [3-5]. All these methods are a combination of PCA and ANOVA. In this paper we will provide a validation procedure for ASCA using a randomization strategy. The models of other ANOVA-based methods may also use this validation procedure.

Analysis of variance simultaneous component analysis, ANOVA-SCA or ASCA is a generalized version of analysis of variance for univariate data to the multivariate case [5]. With this method it is possible to isolate the variation in the data induced by a factor varied in the experimental design. Analyzing this isolated variation with simultaneous component analysis may reveal the relation between the samples and metabolic profile. ASCA successfully helped the quality control in an application of the metabolomics platforms NMR, GC-MS and LC-MS [6]. In an experiment with toxin dosed animals, ASCA successfully disentangled the effects and helped to visualize the homeostatic capacity of the animals [7].

The independent factors in the experimental design translate into a mathematical model that associates the factors to the measured metabolites. It is essential to question whether an effect found in the sample reflects the effect of this specific factor in the population or that it is merely a sampling fluctuation. This paper tries to answers that question and to provide a way to validate ASCA models. Experiments in metabolomics typically have few samples and normality and equal variances can neither be assumed nor tested. Therefore, we propose a procedure for validating megavariate effects in ASCA without the common assumptions of normality or equal variance.

Section two will define the goal and explains some of the theory of statistical validation. That section also explains the ASCA method by defining the model constraints and the used notation scheme. Some of the essential properties, like orthogonality of effect estimates are explained. Explaining ASCA ends with an example of the ASCA model SCA notation. The section that follows details how to randomize the data given the experimental setup of the study. It also details why not to use jackknife or bootstrap, but why permutations are the way to go. A simple example details the model validation, followed by an explanation of how to randomize the data. In section three a simulated data set will serve as an example to certify the validation procedure. Also in that section an experiment with bromobenzene dosed rats will be analyzed and validated. Finally, the last section gives some closing remarks.

## 2 Methods and Theory

### 2.1 Definitions and purpose

The experiments in a metabolomics study often follow an experimental design with varying levels of treatment conditions, also known as factors [8]. Typically the observed metabolic profiles of two different levels of one factor are not the same. This inequality of levels is due to sampling fluctuations and the effect of the varied factor.

### 2.2 ASCA models

This section explains the ANOVA-SCA method. The basis of ASCA is the variation partitioning property of ANOVA that allows estimating the effects of the factors encoded in the experimental design [5]. ASCA has some desirable properties such as orthogonality of effect estimates. Orthogonal effect estimates suit metabolomics experiment analysis well as it allows unique isolation of effect specific variation. Consider, for instance, the case where the treatment regime consists of metabolite data from two dosage levels and three measured time points. ASCA allows isolating the time effects independently from the dosage effects; it can isolate general aging from drug intervention effects.

The variation isolation works as in ANOVA; the preceding example of metabolite data from two dosage levels at three time points translates to a two-way ANOVA design. This design consists of two main effects, time and dosage, and a time dosage interaction effect. The main effects and the interaction effect are all orthogonal; this enables perfect isolation of effect specific variation.

In the following text the boldface uppercase characters represent matrices (**X**), vectors are in lowercase bold-italic (**x**) and scalars in lowercase italic (*x*). The experimental data is shown as **X **(*I *× *J*). The *I *rows contain the samples while the *J *columns in **X **describe the metabolite levels within the samples.

The following text assumes that **X **is mean centered, that is, the mean of each column in **X **is 0, equation 1.

∑i=1Ixij=0∀j
 MathType@MTEF@5@5@+=feaafiart1ev1aaatCvAUfKttLearuWrP9MDH5MBPbIqV92AaeXatLxBI9gBaebbnrfifHhDYfgasaacH8akY=wiFfYdH8Gipec8Eeeu0xXdbba9frFj0=OqFfea0dXdd9vqai=hGuQ8kuc9pgc9s8qqaq=dirpe0xb9q8qiLsFr0=vr0=vr0dc8meaabaqaciaacaGaaeqabaqabeGadaaakeaadaaeWbqaaiabdIha4naaBaaaleaacqWGPbqAcqWGQbGAaeqaaOGaeyypa0JaeGimaaJaeyiaIiIaemOAaOgaleaacqWGPbqAcqGH9aqpcqaIXaqmaeaacqWGjbqsa0GaeyyeIuoaaaa@3BE2@

If the matrix **X**_*δ *_contains the estimates of an effect, then equation 2 defines the sum of squares (SSQ) of that effect, here shown for effect *δ*.

SSQδ=‖Xδ‖2=∑i=1I∑j=1J(xij)2
 MathType@MTEF@5@5@+=feaafiart1ev1aaatCvAUfKttLearuWrP9MDH5MBPbIqV92AaeXatLxBI9gBaebbnrfifHhDYfgasaacH8akY=wiFfYdH8Gipec8Eeeu0xXdbba9frFj0=OqFfea0dXdd9vqai=hGuQ8kuc9pgc9s8qqaq=dirpe0xb9q8qiLsFr0=vr0=vr0dc8meaabaqaciaacaGaaeqabaqabeGadaaakeaacqWGtbWucqWGtbWucqWGrbqudaWgaaWcbaacciGae8hTdqgabeaakiabg2da9maafmaabaacbeGae4hwaG1aaSbaaSqaaiab=r7aKbqabaaakiaawMa7caGLkWoadaahaaWcbeqaaiabikdaYaaakiabg2da9maaqahabaWaaabCaeaacqGGOaakcqWG4baEdaWgaaWcbaGaemyAaKMaemOAaOgabeaakiabcMcaPmaaCaaaleqabaGaeGOmaidaaaqaaiabdQgaQjabg2da9iabigdaXaqaaiabdQeakbqdcqGHris5aaWcbaGaemyAaKMaeyypa0JaeGymaedabaGaemysaKeaniabggHiLdaaaa@5014@

**X**_*τ*_, **X**_*δ *_and **X**_*τδ *_represent the isolated variation due to time, dose, and their interaction respectively. **X**_*e *_contains the individual variation that is not induced by the factors.

A general two-way ANOVA model is shown in equation 3, where *τ *and *δ *are the main effects with levels *c *and *d*, *j *is the variable index. An example of 2-way ANOVA model common in the metabolomics field shows in (equation 4) how the variation is composed of time effects, dosage effects, interaction between time and dosage and residuals (equation 4 and 5). Each of the effect partitions in equation 4 is orthogonal to the others, equation 6. This orthogonal property allows for the variation decomposition shown in equation 4 [5]. The effect estimates are not normalized.

*x*_*cdj *_= *τ*_*cj *_+ *δ*_*dj *_+ (*τδ*)_*cdj*_

Alternatively equation 3 can be written in the matrix form, shown in equation 4.

**X **= **X**_*τ *_+ **X**_*δ *_+ **X**_*τδ *_+ **X**_*e*_

The variation measured in sum of squares, can be uniquely partitioned into the effect, equation 5.

||**X**||^2 ^= ||**X**_*τ*_||^2 ^+ ||**X**_*δ*_||^2 ^+ ||**X**_*τδ*_||^2 ^+||**X**_*e*_||^2^

The orthogonality of the effects is shown in equation 6.

XθTXφ=0∀{θ,φ}⊂{τ,δ,τδ,e}:θ≠φ
 MathType@MTEF@5@5@+=feaafiart1ev1aaatCvAUfKttLearuWrP9MDH5MBPbIqV92AaeXatLxBI9gBaebbnrfifHhDYfgasaacH8akY=wiFfYdH8Gipec8Eeeu0xXdbba9frFj0=OqFfea0dXdd9vqai=hGuQ8kuc9pgc9s8qqaq=dirpe0xb9q8qiLsFr0=vr0=vr0dc8meaabaqaciaacaGaaeqabaqabeGadaaakeaaieqacqWFybawdaqhaaWcbaacciGae4hUdehabaGaemivaqfaaOGae8hwaG1aaSbaaSqaaiab+z8aMbqabaGccqGH9aqpcqaIWaamcqGHaiIicqGG7bWEcqGF4oqCcqGGSaalcqGFgpGzcqGG9bqFcqGHckcZcqGG7bWEcqGFepaDcqGGSaalcqGF0oazcqGGSaalcqGFepaDcqGF0oazcqGGSaalcqWGLbqzcqGG9bqFcqGG6aGocqGF4oqCcqGHGjsUcqGFgpGzaaa@5405@

The SCA estimates the information in the partitions time, dosage and dosage time interaction. The two-way ANOVA style ASCA model (equations 3, 4) give the following ASCA model after SCA (equation 7):

X=TτPτt+TδPδT+T(τδ)P(τδ)T+TePeT+E
 MathType@MTEF@5@5@+=feaafiart1ev1aaatCvAUfKttLearuWrP9MDH5MBPbIqV92AaeXatLxBI9gBaebbnrfifHhDYfgasaacH8akY=wiFfYdH8Gipec8Eeeu0xXdbba9frFj0=OqFfea0dXdd9vqai=hGuQ8kuc9pgc9s8qqaq=dirpe0xb9q8qiLsFr0=vr0=vr0dc8meaabaqaciaacaGaaeqabaqabeGadaaakeaaieqacqWFybawcqGH9aqpcqWFubavdaWgaaWcbaacciGae4hXdqhabeaakiab=bfaqnaaDaaaleaacqGFepaDaeaacqWG0baDaaGccqGHRaWkcqWFubavdaWgaaWcbaGae4hTdqgabeaakiab=bfaqnaaDaaaleaacqGF0oazaeaacqWGubavaaGccqGHRaWkcqWFubavdaWgaaWcbaGaeiikaGIae4hXdqNae4hTdqMaeiykaKcabeaakiab=bfaqnaaDaaaleaacqGGOaakcqGFepaDcqGF0oazcqGGPaqkaeaacqWGubavaaGccqGHRaWkcqWFubavdaWgaaWcbaGaemyzaugabeaakiab=bfaqnaaDaaaleaacqWGLbqzaeaacqWGubavaaGccqGHRaWkcqWGfbqraaa@571A@

A more detailed review of the ASCA method properties is found elsewhere [9].

### 2.3 Type of resampling to use

A way to tackle the problem of validating ASCA models is by using resampling techniques, being jackknife, bootstrapping and permutation tests [10]. The basic idea will be explained by a univariate analysis of two groups of equal size. Later, this will be generalized to the megavariate case. The standard way of testing the difference between group means, with the underlying null-hypothesis that the population group means are not different, is with a t-test. The ANOVA F-test comes down to a t-test for the two group case. Under the assumptions of normality and equal group variances the t-statistic is

t=(x¯1−x¯2)sp
 MathType@MTEF@5@5@+=feaafiart1ev1aaatCvAUfKttLearuWrP9MDH5MBPbIqV92AaeXatLxBI9gBaebbnrfifHhDYfgasaacH8akY=wiFfYdH8Gipec8Eeeu0xXdbba9frFj0=OqFfea0dXdd9vqai=hGuQ8kuc9pgc9s8qqaq=dirpe0xb9q8qiLsFr0=vr0=vr0dc8meaabaqaciaacaGaaeqabaqabeGadaaakeaacqWG0baDcqGH9aqpdaWcaaqaaiabcIcaOiqbdIha4zaaraWaaSbaaSqaaiabigdaXaqabaGccqGHsislcuWG4baEgaqeamaaBaaaleaacqaIYaGmaeqaaOGaeiykaKcabaGaem4Cam3aaSbaaSqaaiabdchaWbqabaaaaaaa@3A46@

sp=1n(s12+s22)
 MathType@MTEF@5@5@+=feaafiart1ev1aaatCvAUfKttLearuWrP9MDH5MBPbIqV92AaeXatLxBI9gBaebbnrfifHhDYfgasaacH8akY=wiFfYdH8Gipec8Eeeu0xXdbba9frFj0=OqFfea0dXdd9vqai=hGuQ8kuc9pgc9s8qqaq=dirpe0xb9q8qiLsFr0=vr0=vr0dc8meaabaqaciaacaGaaeqabaqabeGadaaakeaacqWGZbWCdaWgaaWcbaGaemiCaahabeaakiabg2da9maakaaabaWaaSaaaeaacqaIXaqmaeaacqWGUbGBaaGaeiikaGIaem4Cam3aa0baaSqaaiabigdaXaqaaiabikdaYaaakiabgUcaRiabdohaZnaaDaaaleaacqaIYaGmaeaacqaIYaGmaaGccqGGPaqkaSqabaaaaa@3CE6@

where x¯1
 MathType@MTEF@5@5@+=feaafiart1ev1aaatCvAUfKttLearuWrP9MDH5MBPbIqV92AaeXatLxBI9gBaebbnrfifHhDYfgasaacH8akY=wiFfYdH8Gipec8Eeeu0xXdbba9frFj0=OqFfea0dXdd9vqai=hGuQ8kuc9pgc9s8qqaq=dirpe0xb9q8qiLsFr0=vr0=vr0dc8meaabaqaciaacaGaaeqabaqabeGadaaakeaacuWG4baEgaqeamaaBaaaleaacqaIXaqmaeqaaaaa@2F59@ and x¯2
 MathType@MTEF@5@5@+=feaafiart1ev1aaatCvAUfKttLearuWrP9MDH5MBPbIqV92AaeXatLxBI9gBaebbnrfifHhDYfgasaacH8akY=wiFfYdH8Gipec8Eeeu0xXdbba9frFj0=OqFfea0dXdd9vqai=hGuQ8kuc9pgc9s8qqaq=dirpe0xb9q8qiLsFr0=vr0=vr0dc8meaabaqaciaacaGaaeqabaqabeGadaaakeaacuWG4baEgaqeamaaBaaaleaacqaIYaGmaeqaaaaa@2F5B@ are the group means, *n *the num-ber of samples in the groups and *s*_1 _and *s*_2 _are the group standard deviations [11]. The pooled standard deviation *s*_*p *_can be calculated easily from *s*_1 _and *s*_2 _given the assumptions of normality and equal group variances. Actually, *s*_*p *_is the standard deviation of (x¯1
 MathType@MTEF@5@5@+=feaafiart1ev1aaatCvAUfKttLearuWrP9MDH5MBPbIqV92AaeXatLxBI9gBaebbnrfifHhDYfgasaacH8akY=wiFfYdH8Gipec8Eeeu0xXdbba9frFj0=OqFfea0dXdd9vqai=hGuQ8kuc9pgc9s8qqaq=dirpe0xb9q8qiLsFr0=vr0=vr0dc8meaabaqaciaacaGaaeqabaqabeGadaaakeaacuWG4baEgaqeamaaBaaaleaacqaIXaqmaeqaaaaa@2F59@ −  x¯2
 MathType@MTEF@5@5@+=feaafiart1ev1aaatCvAUfKttLearuWrP9MDH5MBPbIqV92AaeXatLxBI9gBaebbnrfifHhDYfgasaacH8akY=wiFfYdH8Gipec8Eeeu0xXdbba9frFj0=OqFfea0dXdd9vqai=hGuQ8kuc9pgc9s8qqaq=dirpe0xb9q8qiLsFr0=vr0=vr0dc8meaabaqaciaacaGaaeqabaqabeGadaaakeaacuWG4baEgaqeamaaBaaaleaacqaIYaGmaeqaaaaa@2F5B@), showing the rationale of the t-statistic: a measure of the devia-tion (x¯1
 MathType@MTEF@5@5@+=feaafiart1ev1aaatCvAUfKttLearuWrP9MDH5MBPbIqV92AaeXatLxBI9gBaebbnrfifHhDYfgasaacH8akY=wiFfYdH8Gipec8Eeeu0xXdbba9frFj0=OqFfea0dXdd9vqai=hGuQ8kuc9pgc9s8qqaq=dirpe0xb9q8qiLsFr0=vr0=vr0dc8meaabaqaciaacaGaaeqabaqabeGadaaakeaacuWG4baEgaqeamaaBaaaleaacqaIXaqmaeqaaaaa@2F59@ −  x¯2
 MathType@MTEF@5@5@+=feaafiart1ev1aaatCvAUfKttLearuWrP9MDH5MBPbIqV92AaeXatLxBI9gBaebbnrfifHhDYfgasaacH8akY=wiFfYdH8Gipec8Eeeu0xXdbba9frFj0=OqFfea0dXdd9vqai=hGuQ8kuc9pgc9s8qqaq=dirpe0xb9q8qiLsFr0=vr0=vr0dc8meaabaqaciaacaGaaeqabaqabeGadaaakeaacuWG4baEgaqeamaaBaaaleaacqaIYaGmaeqaaaaa@2F5B@) in its standard deviation units *s*_*p*_. Including the proper degrees of freedom allows for testing the null-hypothesis of equal group means.

The bootstrap and jackknife work by resampling the samples in the groups, keeping the grouping structure intact, estimating from those resamplings the group standard deviations *s*_1 _and *s*_2_. However, this does not directly give the wanted result, because the value needed for the t-test is the standard deviation of (x¯1
 MathType@MTEF@5@5@+=feaafiart1ev1aaatCvAUfKttLearuWrP9MDH5MBPbIqV92AaeXatLxBI9gBaebbnrfifHhDYfgasaacH8akY=wiFfYdH8Gipec8Eeeu0xXdbba9frFj0=OqFfea0dXdd9vqai=hGuQ8kuc9pgc9s8qqaq=dirpe0xb9q8qiLsFr0=vr0=vr0dc8meaabaqaciaacaGaaeqabaqabeGadaaakeaacuWG4baEgaqeamaaBaaaleaacqaIXaqmaeqaaaaa@2F59@ −  x¯2
 MathType@MTEF@5@5@+=feaafiart1ev1aaatCvAUfKttLearuWrP9MDH5MBPbIqV92AaeXatLxBI9gBaebbnrfifHhDYfgasaacH8akY=wiFfYdH8Gipec8Eeeu0xXdbba9frFj0=OqFfea0dXdd9vqai=hGuQ8kuc9pgc9s8qqaq=dirpe0xb9q8qiLsFr0=vr0=vr0dc8meaabaqaciaacaGaaeqabaqabeGadaaakeaacuWG4baEgaqeamaaBaaaleaacqaIYaGmaeqaaaaa@2F5B@). Assuming normality and equal variances, this value can be calculated from the group variances using equation 9. This is a reasonable assumption for analytical replicates of a sample, but not directly for the biological variation across subjects. The assumption of equal group variances is questionable in this case. These assumptions cannot easily be tested given the small group sample sizes. Thus, it is not clear how to obtain a standard deviation value for (x¯1
 MathType@MTEF@5@5@+=feaafiart1ev1aaatCvAUfKttLearuWrP9MDH5MBPbIqV92AaeXatLxBI9gBaebbnrfifHhDYfgasaacH8akY=wiFfYdH8Gipec8Eeeu0xXdbba9frFj0=OqFfea0dXdd9vqai=hGuQ8kuc9pgc9s8qqaq=dirpe0xb9q8qiLsFr0=vr0=vr0dc8meaabaqaciaacaGaaeqabaqabeGadaaakeaacuWG4baEgaqeamaaBaaaleaacqaIXaqmaeqaaaaa@2F59@ −  x¯2
 MathType@MTEF@5@5@+=feaafiart1ev1aaatCvAUfKttLearuWrP9MDH5MBPbIqV92AaeXatLxBI9gBaebbnrfifHhDYfgasaacH8akY=wiFfYdH8Gipec8Eeeu0xXdbba9frFj0=OqFfea0dXdd9vqai=hGuQ8kuc9pgc9s8qqaq=dirpe0xb9q8qiLsFr0=vr0=vr0dc8meaabaqaciaacaGaaeqabaqabeGadaaakeaacuWG4baEgaqeamaaBaaaleaacqaIYaGmaeqaaaaa@2F5B@) from the jackknifed or bootstrapped *s*_1 _and *s*_2 _without making extra assumptions.

Permutation tests work directly on the variability of (x¯1
 MathType@MTEF@5@5@+=feaafiart1ev1aaatCvAUfKttLearuWrP9MDH5MBPbIqV92AaeXatLxBI9gBaebbnrfifHhDYfgasaacH8akY=wiFfYdH8Gipec8Eeeu0xXdbba9frFj0=OqFfea0dXdd9vqai=hGuQ8kuc9pgc9s8qqaq=dirpe0xb9q8qiLsFr0=vr0=vr0dc8meaabaqaciaacaGaaeqabaqabeGadaaakeaacuWG4baEgaqeamaaBaaaleaacqaIXaqmaeqaaaaa@2F59@ −  x¯2
 MathType@MTEF@5@5@+=feaafiart1ev1aaatCvAUfKttLearuWrP9MDH5MBPbIqV92AaeXatLxBI9gBaebbnrfifHhDYfgasaacH8akY=wiFfYdH8Gipec8Eeeu0xXdbba9frFj0=OqFfea0dXdd9vqai=hGuQ8kuc9pgc9s8qqaq=dirpe0xb9q8qiLsFr0=vr0=vr0dc8meaabaqaciaacaGaaeqabaqabeGadaaakeaacuWG4baEgaqeamaaBaaaleaacqaIYaGmaeqaaaaa@2F5B@) by randomly permuting class labels and recalculating the group-mean differences. Actually, such permutation tests go back a long way [12] as an alternative for t-tests and are now also routinely used in gene-expression data analysis, as for instance Significance Analysis of Microarrays (SAM) [13].

The standard deviation of (x¯1
 MathType@MTEF@5@5@+=feaafiart1ev1aaatCvAUfKttLearuWrP9MDH5MBPbIqV92AaeXatLxBI9gBaebbnrfifHhDYfgasaacH8akY=wiFfYdH8Gipec8Eeeu0xXdbba9frFj0=OqFfea0dXdd9vqai=hGuQ8kuc9pgc9s8qqaq=dirpe0xb9q8qiLsFr0=vr0=vr0dc8meaabaqaciaacaGaaeqabaqabeGadaaakeaacuWG4baEgaqeamaaBaaaleaacqaIXaqmaeqaaaaa@2F59@ −  x¯2
 MathType@MTEF@5@5@+=feaafiart1ev1aaatCvAUfKttLearuWrP9MDH5MBPbIqV92AaeXatLxBI9gBaebbnrfifHhDYfgasaacH8akY=wiFfYdH8Gipec8Eeeu0xXdbba9frFj0=OqFfea0dXdd9vqai=hGuQ8kuc9pgc9s8qqaq=dirpe0xb9q8qiLsFr0=vr0=vr0dc8meaabaqaciaacaGaaeqabaqabeGadaaakeaacuWG4baEgaqeamaaBaaaleaacqaIYaGmaeqaaaaa@2F5B@) has the squared Euclidean distance in its numerator, the denominator is constant over the permutations. In centered data the squared Euclidean distance equals the sum of squares (SSQ) of (x¯1
 MathType@MTEF@5@5@+=feaafiart1ev1aaatCvAUfKttLearuWrP9MDH5MBPbIqV92AaeXatLxBI9gBaebbnrfifHhDYfgasaacH8akY=wiFfYdH8Gipec8Eeeu0xXdbba9frFj0=OqFfea0dXdd9vqai=hGuQ8kuc9pgc9s8qqaq=dirpe0xb9q8qiLsFr0=vr0=vr0dc8meaabaqaciaacaGaaeqabaqabeGadaaakeaacuWG4baEgaqeamaaBaaaleaacqaIXaqmaeqaaaaa@2F59@ −  x¯2
 MathType@MTEF@5@5@+=feaafiart1ev1aaatCvAUfKttLearuWrP9MDH5MBPbIqV92AaeXatLxBI9gBaebbnrfifHhDYfgasaacH8akY=wiFfYdH8Gipec8Eeeu0xXdbba9frFj0=OqFfea0dXdd9vqai=hGuQ8kuc9pgc9s8qqaq=dirpe0xb9q8qiLsFr0=vr0=vr0dc8meaabaqaciaacaGaaeqabaqabeGadaaakeaacuWG4baEgaqeamaaBaaaleaacqaIYaGmaeqaaaaa@2F5B@). Using the SSQ as effect statistic, the generalization from univariate to multivariate follows from summing the univariate SSQ's for all variables.

### 2.4 How to randomize the data

Randomizing or permutation is the uncoupling of the data from the group labels [14,15]. Take note that in data with a zero mean (equation 1) the random sampling expectated value is 0. Considering the level averages, the randomization procedure tests whether the results with randomized labels are as different from zero as the original result is. The randomization, or permutation, does not change the metabolite values for a sample, but it reassigns each sample randomly to one of the treatment groups.

### 2.5 Model validation example

This section gives a detailed example of how the permutation works and how it will help to validate models.

In most experimental designs it is important to assess the statistical confidence of the effect estimates. An experiment with two measurement series and three measurements in each series will serve as example for the validation. If these series are ***a ***and ***b***, the two series comprise the levels of the effect *δ*, giving the model shown *d *in equation 10. This equation holds for both vectors and matrices, shown here is the matrix form of effect of factor *δ*. The null hypothesis (*H*_0_) is the sum-of-squares (SSQ) associated with the effect of factor *δ *is zero (Equation 11). The alternative hypothesis (*H*_1_) states the that SSQ of the effect of factor *δ *is larger than zero.

**X **= **X**_*δ *_+ **X**_*e*_

*H*_0 _: ||**X**_*δ*_||^2 ^= 0;*H*_1 _: ||**X**_*δ*_||^2 ^> 0

The chosen distance measure that marks how far the group averages are apart is the squared Euclidean distance. In the hypothetical case with a known population, the factors without effect have an SSQ that is zero. Due to the small sample size, the distance between ***a ***and ***b ***will never be exactly zero, giving an ||**X**_*δ*_||^2 ^*> *0. The SSQ is by its nature also a distance measure that describes how far the effect levels are from zero. In the univariate context, usually variances of the groups are analyzed. In the multivariate context the analysis focuses on SSQ's. The SSQ also conveniently describes the variation in the data.

The measured results for series ***a ***are 5, 4 and 3, for series ***b ***the results are -3, -4 and -5. These values satisfy equation 1. The average of level ***a ***is 4 and the average of level ***b ***is -4, shown in equation 12.

x=[ab]=[543−3−4−5],xδ=[a¯a¯a¯b¯b¯b¯],xδ=[444−4−4−4]
 MathType@MTEF@5@5@+=feaafiart1ev1aaatCvAUfKttLearuWrP9MDH5MBPbIqV92AaeXatLxBI9gBaebbnrfifHhDYfgasaacH8akY=wiFfYdH8Gipec8Eeeu0xXdbba9frFj0=OqFfea0dXdd9vqai=hGuQ8kuc9pgc9s8qqaq=dirpe0xb9q8qiLsFr0=vr0=vr0dc8meaabaqaciaacaGaaeqabaqabeGadaaakeaaieWacqWF4baEcqGH9aqpdaWadaqaauaabeqaceaaaeaacqWFHbqyaeaacqWFIbGyaaaacaGLBbGaayzxaaGaeyypa0ZaamWaaeaafaqaceGbbaaaaeaacqaI1aqnaeaacqaI0aanaeaacqaIZaWmaeaacqGHsislcqaIZaWmaeaacqGHsislcqaI0aanaeaacqGHsislcqaI1aqnaaaacaGLBbGaayzxaaGaeiilaWIae8hEaG3aaSbaaSqaaGGaciab+r7aKbqabaGccqGH9aqpdaWadaqaauaabeqageaaaaqaaiqb=fgaHzaaraaabaGaf8xyaeMbaebaaeaacuWFHbqygaqeaaqaaiqb=jgaIzaaraaabaGaf8NyaiMbaebaaeaacuWFIbGygaqeaaaaaiaawUfacaGLDbaacqGGSaalcqWF4baEdaWgaaWcbaGae4hTdqgabeaakiabg2da9maadmaabaqbaeGabyqaaaaabaGaeGinaqdabaGaeGinaqdabaGaeGinaqdabaGaeyOeI0IaeGinaqdabaGaeyOeI0IaeGinaqdabaGaeyOeI0IaeGinaqdaaaGaay5waiaaw2faaaaa@5EA1@

Randomization is uncoupling the group labels from the data and randomly reassigning them. To show the randomization the samples with the ± 3 will switch groups. Level ***a ***now has the measured values -3, 4 and 5, while level ***b ***has 3, -4 and -5, equation 13. *x*_*p *_shows the permuted ***x ***and xδp
 MathType@MTEF@5@5@+=feaafiart1ev1aaatCvAUfKttLearuWrP9MDH5MBPbIqV92AaeXatLxBI9gBaebbnrfifHhDYfgasaacH8akY=wiFfYdH8Gipec8Eeeu0xXdbba9frFj0=OqFfea0dXdd9vqai=hGuQ8kuc9pgc9s8qqaq=dirpe0xb9q8qiLsFr0=vr0=vr0dc8meaabaqaciaacaGaaeqabaqabeGadaaakeaaieWacqWF4baEdaqhaaWcbaacciGae4hTdqgabaGaemiCaahaaaaa@316E@ its average.

xp=[54−33−4−5],xδp=[222−2−2−2]
 MathType@MTEF@5@5@+=feaafiart1ev1aaatCvAUfKttLearuWrP9MDH5MBPbIqV92AaeXatLxBI9gBaebbnrfifHhDYfgasaacH8akY=wiFfYdH8Gipec8Eeeu0xXdbba9frFj0=OqFfea0dXdd9vqai=hGuQ8kuc9pgc9s8qqaq=dirpe0xb9q8qiLsFr0=vr0=vr0dc8meaabaqaciaacaGaaeqabaqabeGadaaakeaaieWacqWF4baEdaahaaWcbeqaaiabdchaWbaakiabg2da9maadmaabaqbaeGabyqaaaaabaGaeGynaudabaGaeGinaqdabaacceGae4NeI0ccbeGae03mamdabaGae03mamdabaGaeyOeI0IaeGinaqdabaGaeyOeI0IaeGynaudaaaGaay5waiaaw2faaiabcYcaSiab=Hha4naaDaaaleaaiiGacqaF0oazaeaacqWGWbaCaaGccqGH9aqpdaWadaqaauaabiqageaaaaqaaiabikdaYaqaaiabikdaYaqaaiabikdaYaqaaiabgkHiTiabikdaYaqaaiabgkHiTiabikdaYaqaaiabgkHiTiabikdaYaaaaiaawUfacaGLDbaaaaa@4C7C@

The distance between the averages is much smaller in the randomized set than the averages of the original data, equation 14.

‖xδ‖2=6∗(±4)2=93 and ‖xδp‖2=6∗(±2)2=24
 MathType@MTEF@5@5@+=feaafiart1ev1aaatCvAUfKttLearuWrP9MDH5MBPbIqV92AaeXatLxBI9gBaebbnrfifHhDYfgasaacH8akY=wiFfYdH8Gipec8Eeeu0xXdbba9frFj0=OqFfea0dXdd9vqai=hGuQ8kuc9pgc9s8qqaq=dirpe0xb9q8qiLsFr0=vr0=vr0dc8meaabaqaciaacaGaaeqabaqabeGadaaakeaadaqbdaqaaGqadiab=Hha4naaBaaaleaaiiGacqGF0oazaeqaaaGccaGLjWUaayPcSdWaaWbaaSqabeaacqaIYaGmaaGccqGH9aqpcqaI2aGncqGHxiIkcqGGOaakcqGHXcqScqaI0aancqGGPaqkdaahaaWcbeqaaiabikdaYaaakiabg2da9iabiMda5iabiodaZiabbccaGiabbggaHjabb6gaUjabbsgaKjabbccaGmaafmaabaGae8hEaG3aa0baaSqaaiab+r7aKbqaaiabdchaWbaaaOGaayzcSlaawQa7amaaCaaaleqabaGaeGOmaidaaOGaeyypa0JaeGOnayJaey4fIOIaeiikaGIaeyySaeRaeGOmaiJaeiykaKYaaWbaaSqabeaacqaIYaGmaaGccqGH9aqpcqaIYaGmcqaI0aanaaa@5A2B@

The distance between series ***a ***and ***b ***is much larger than any of the SSQ's after randomization. There is no permutation that gives a larger SSQ than the original grouping. The larger distance in the original model suggests a significant difference in the series ***a ***and ***b***, equation 15.

‖xδp‖2≪‖xδ‖2⇒H0 probably false
 MathType@MTEF@5@5@+=feaafiart1ev1aaatCvAUfKttLearuWrP9MDH5MBPbIqV92AaeXatLxBI9gBaebbnrfifHhDYfgasaacH8akY=wiFfYdH8Gipec8Eeeu0xXdbba9frFj0=OqFfea0dXdd9vqai=hGuQ8kuc9pgc9s8qqaq=dirpe0xb9q8qiLsFr0=vr0=vr0dc8meaabaqaciaacaGaaeqabaqabeGadaaakeaadaqbdaqaaGqadiab=Hha4naaDaaaleaaiiGacqGF0oazaeaacqWGWbaCaaaakiaawMa7caGLkWoadaahaaWcbeqaaiabikdaYaaakiablQMi9maafmaabaGae8hEaG3aaSbaaSqaaiab+r7aKbqabaaakiaawMa7caGLkWoadaahaaWcbeqaaiabikdaYaaakiabgkDiElabdIeainaaBaaaleaacqaIWaamaeqaaOGaeeiiaaIaeeiCaaNaeeOCaiNaee4Ba8MaeeOyaiMaeeyyaeMaeeOyaiMaeeiBaWMaeeyEaKNaeeiiaaIaeeOzayMaeeyyaeMaeeiBaWMaee4CamNaeeyzaugaaa@568B@

Randomly reassigning multivariate samples to a group works in the same way as described in the preceding paragraphs for univariate data. The randomization leaves the order of metabolites of the sample unaffected. The SSQ, equation 2, allows for univariate and multivariate calculation of the sum of squares; thereby forming the generalization to the multivariate case.

Repeating the randomization procedure many times gives just as many SSQ values. These values define the reference distribution. When most of the randomization SSQ results are larger than the original group assignment result, the effect SSQ is a sampling fluctuations and *H*_0 _is not to be rejected. Finally, the probability value or p-value is defined to be the number of SSQs in the reference distribution that is larger than the original SSQ. So when 35 of the 1000 SSQs in the reference distribution are larger than the original SSQ, the probability of finding a larger than original SSQ value is (p-value) 35/1000 = 0.035.

A good estimate on the randomized SSQ distribution needs many randomization iterations. How many randomization iterations are enough for a good probability estimate is difficult to establish before starting, because that largely depends on the data. However, repeating the randomization series should give similar results, this suggests enough iteration. The permutations are a random subset of all the possible permutations [15]. This approach is also known as Monte Carlo resampling.

This method validates the multivariate ANOVA partitioning, not the SCA part of ASCA. The SCA method subsequently describes most variation within each partition.

### 2.6 One-Way ANOVA Design

The preceding example is an example of a one-way ANOVA design with two levels (equation 12). To get a reference distribution one can simply permute the group labels. If the original SSQ is larger than most of the reference distribution the model is considered significant, otherwise it is not. In the preceding example series ***a ***is significantly different from series ***b***.

### 2.7 Two-Way ANOVA Design

A two-way ANOVA extends the one-way ANOVA design to two factors. In metabolomics experiments common factor examples are time and a drug intervention.

Unlike one-way designs two-way ANOVA designs may also include an interaction term. The interaction captures the relation between two main factors. In a time and drug example, the interaction effect means the drug shows a different at different time points.

Each main effect needs to be validated separately, getting the reference distribution for the main effect is the same as for the one-way ANOVA. Getting the reference distribution for the interaction term is a bit more complicated. The best option is to permute the residual samples (equation 16) [14]. Residual samples are samples that have the main effects removed.

**X**_*r *_= **X **- **X**_*τ *_- **X**_*δ*_

### 2.8 Nested ANOVA Design

Nested ANOVA designs are extensions of ANOVA design with another factor nested in the main effect. Some special cases need nested ANOVA models, like experiments that measure one animal at different times. The repeated measuring nests the factor time in the animal. The randomization strategy in such cases only allows for placing the animal time series in other levels of the one-way ANOVA factor. In a nested ANOVA design, the permutable unit is the animal itself [2,14,15].

### 2.9 Related Methods

A widely used method in metabolomics is principal component analysis. This method, however, does not take group structures into account, hindering the analysis of effects. Methods that are more closely related to ASCA are SMART and PRC [4,16]. However, these methods differ on a key issue, namely orthogonality of effect estimates (equation 5). The effect estimates of SMART are not orthogonal, as a result the here proposed validation procedure cannot be used. In PRC the effect estimates are orthogonal up to the deflation of the control condition. The proposed validation procedure can be used in PRC as long as it is used before deflating the control effect.

### 2.10 Experimental environment

The ASCA algorithm was implemented in MATLAB script code, using The MathWorks MATLAB version 7.1 release 14 running on Fedora Core 3 on an Intel Corporation Pentium IV (3.0 GHz) computer.

The ASCA algorithm is in the download section of our website [17]. The validation algorithm can be found there as well.

## 3 Results & Discussion

This section shows results to certify the proposed validation method for synthetic data and real world experimental data.

The real world experiment deals with toxin-dosed rats [7]. Various other methods already analyzed this experiment; the results strongly suggest the toxin is affecting the animal. This experiment serves as a real world certification of the suggested statistical validation approach in multivariate data sets.

### 3.1 Examples; certifying the procedures with designed data

This example study showcases two data sets. The first data set has two effect levels that are significantly different. The second data set has two effect levels that are not significantly different. This is to test the suggested procedure in the simple case with known true statistics.

ASCA describes each effect level by the averages of the metabolites in that level. In this example study, ASCA will test if the multivariate average of the first 10 rows is different from the multivariate average of the last 10 rows. When many randomizations give an SSQ that is equally large as the original SSQ, the groups probably do not differ. When only a minor fraction of the randomizations give a larger group distance, the groups most likely differ.

In the model the effect *δ *has two levels (equation 10). *d *In the first data set the first 10 rows are filled with ones and the last 10 rows are filled with zeros. Normal distributed white noise (*N*(*σ *= 1, *μ *= 0)) is added to this data. The second data set is filled with zeros and white noise (*N*(*σ *= 1, *μ *= 0)) is added to it.

**Figure 1:**
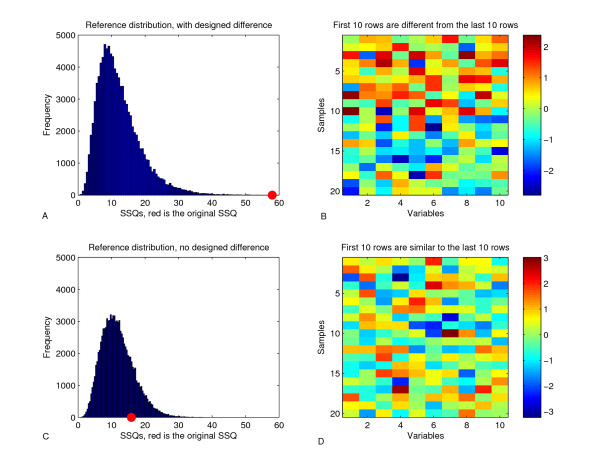
**Example study to certify the validation procedure, it consists of one significantly different and one nonsignificantly different data set**. Figures A and C show the SSQ reference distribution found by permuting the data. If the red dot is outside most the reference distribution and is on the right side, the group is significantly different. The figures B and D show the data from this example experiment. Careful inspection of figure B reveals the top half differs from the bottom half, it is more yellow and red then the bottom half. The D figure lacks this property.

Figures 1b &1d show the two example data sets, the rows are individual samples and the columns are the metabolites. The colored cells show each metabolite value of every sample.

In the true significant example the effect *δ *is designed to be different. The top half of figure 1b has more red colored cells while the bottom half has more blue colored cells. Figure 1a shows the reference distribution of randomized SSQ's, using a vertical line to show the SSQ of the original grouping.

Following the proposed validation procedure, the conclusion is clear: the halves are unlikely to be the same because all the permuted SSQ's are smaller than the original SSQ (p = 0.00012, SSQ = 57.96). Conclusion: the difference in levels is significant. This model validation used 100,000 randomization iterations taking about 5 minutes of computing time.

Repeating the validation procedure on data without a designed difference between the two dosage levels, serves as a negative control. The level averages will differ a little, but these differences are sampling fluctuations.

Figure 1d is similar to figure 1b but without the designed differences in the dosage levels. Figure 1d does not show a seeming difference between the top and bottom half. Figure 1c shows the reference distribution for the data set equal level averages.

Following the proposed validation procedure, the conclusion is clear: the halves are likely to be the same because many (19.46%) of the permuted SSQ's are larger than the original SSQ (p = 0.19463, SSQ = 15.91). Conclusion: the difference in levels is not significant. This model validation used 100,000 randomization iterations taking about 5 minutes of computing time.

To test if the proposed validation procedure rejects the *H*_0 _in the fraction of the significance threshold, the model from equation 10 was used with 1000 different realisations of white noise (*σ *= 1, *μ *= 0). With an significance threshold of *α *= 0.05, 50 of the 1000 *H*_0_'s are expected to be rejected. The number of rejections were in the expected range, given a 95% confidence interval from a binomial distribution with *α *= 0.05 for n = 1000.

### 3.2 Experimental results: Rats dosed with hep-atotoxicant bromobenzene

In this experiment there are five groups of rats; a control group, a corn oil (the toxin vehicle) control group and a low, medium and high dosage of bromobenzene. The collected urine from three individual rats of each treatment group is measured on the NRM platform, at 6, 24 and 48 hours after the toxin administration [7,18]. The rats are sacrificed after each sampling to collect tissue sample for histology and transcriptomics analysis.

One sample from the highest dosage group is missing. To avoid unbalanced ANOVA issues we assume this missing sample equals the average of the two samples collected and measured from that group at the same time point.

**Figure 2:**
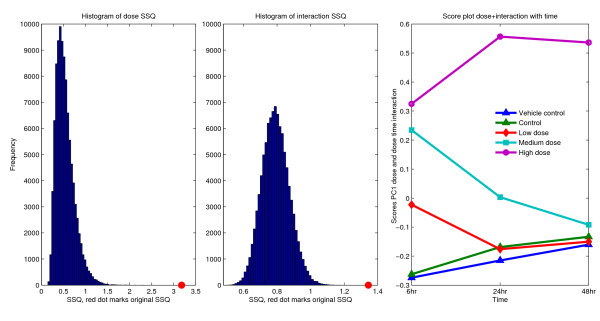
**Validation of the ASCA model for bromobenzene treated rats, validation of the dosage and the dosage-time interaction and the **X**_*δ *_+ **X**_*τδ *_score plot**. This experiment deals with the urine analysis of bromobenzene treated rats, the experimental design includes two types of controls and 3 dosage levels of the hepatotoxicant bromobenzene. The dosage and the interaction models are both significant as is clear from the reference distributions (p ≤ 0.0001). Because the dosage and the interaction models are significant they are superimposed and analyzed by SCA. The score plot of the SCA solution is shown. From this plot it is clear by visual inspection that the average dosage levels differ and that the interaction effect exists.

The main effects and the interaction effect of the 2-way ANOVA models were tested by the ASCA validation. Here the focus is on the factor dosage and dosage-time interaction. The models are significant, with a drug dose difference p ≤ 0.0001, SSQ = 3.181 (figure 2a) and dosage-time interaction p ≤ 0.0001, SSQ = 1.344 (figure 2b). The interaction significance was calculated on the residuals, thus after removing the time and dosage effect (equation 16). The not nested experimental design allows the use of a simple two-way ANOVA permutation scheme.

The dosage and the interaction effect are significant, combining the dosage and interaction gives a data set that describes all effects that depend on dosage (equation 17). SCA helps to reduce the dimensionality of this data set.

**X**_*δ*+*τδ *_= **X**_*δ *_+ **X**_*τδ*_

Xδ+τδ=T(δ+τδ)P(δ+τδ)T+E
 MathType@MTEF@5@5@+=feaafiart1ev1aaatCvAUfKttLearuWrP9MDH5MBPbIqV92AaeXatLxBI9gBaebbnrfifHhDYfgasaacH8akY=wiFfYdH8Gipec8Eeeu0xXdbba9frFj0=OqFfea0dXdd9vqai=hGuQ8kuc9pgc9s8qqaq=dirpe0xb9q8qiLsFr0=vr0=vr0dc8meaabaqaciaacaGaaeqabaqabeGadaaakeaaieqacqWFybawdaWgaaWcbaacciGae4hTdqMaey4kaSIae4hXdqNae4hTdqgabeaakiabg2da9iab=rfaunaaBaaaleaacqGGOaakcqGF0oazcqGHRaWkcqGFepaDcqGF0oazcqGGPaqkaeqaaOGae8huaa1aa0baaSqaaiabcIcaOiab+r7aKjabgUcaRiab+r8a0jab+r7aKjabcMcaPaqaaiabdsfaubaakiabgUcaRiab=veafbaa@4A15@

SCA summarizes the validated toxin and interaction variation. Grouping the scores (*T *in equation 18) according to the factor levels gives figure 2c. The conclusion is the treatment with the hepatotoxicant differs between dosage groups and the dosage responses change over time. Additionally, the results suggest the animals treated with the lowest dosage fully recover or go back to the state of the controls. The animals dosed with the medium dosage need more time, but also go back to the control state. The animals given the highest dosage do not recover to the control state. Histological liver examination revealed extensive damage caused by the bro-mobenzene, corroborating these findings.

## 4 Conclusion

Extending ASCA with a permutation procedure enables validation of ASCA models. Referencing the ASCA models to the permutation based reference distribution gives validation statistics. If the model is significant, the following SCA decomposition describes the validated induced effects.

The proposed method gives validation statistics to the ASCA models. ASCA itself allows for summarizing the designed experimental data. Combining ASCA and the ASCA model validation forms a powerful summary of designed experimental data.
